# Extensive Atrophic Gastritis Increases Intraduodenal Hydrogen Gas

**DOI:** 10.1155/2008/584929

**Published:** 2008-06-16

**Authors:** Yoshihisa Urita, Toshiyasu Watanabe, Tadashi Maeda, Tomohiro Arita, Yosuke Sasaki, Takamasa Ishii, Tatsuhiro Yamamoto, Akiro Kugahara, Asuka Nakayama, Makie Nanami, Kaoru Domon, Susumu Ishihara, Hirohito Kato, Kazuo Hike, Norikok Hara, Shuji Watanabe, Kazushige Nakanishi, Motonobu Sugimoto, Kazumasa Miki

**Affiliations:** ^1^Department of General Medicine and Emergency Care, School of Medicine, Toho University, Omori Hospital, Tokyo 143-8541, Japan; ^2^Department of Hematology, School of Medicine, Toho University, Omori Hospital, Tokyo 143-8541, Japan; ^3^Division of Gastroenterology and Hepatology, School of Medicine, Toho University, Omori Hospital, Tokyo 143-8541, Japan; ^4^Department of Internal Medicine, School of Medicine, Toho University, Omori Hospital, Tokyo 143-8541, Japan

## Abstract

*Objective*. Gastric acid plays an important part in the prevention of bacterial colonization of the gastrointestinal tract. If these bacteria have an ability of hydrogen (H2) fermentation, intraluminal H2 gas might be detected. We attempted to measure the intraluminal H2 concentrations to determine the bacterial overgrowth in the gastrointestinal tract. *Patients and methods*. Studies were performed in 647 consecutive patients undergoing upper endoscopy. At the time of endoscopic examination, we intubated the stomach and the descending part of the duodenum without inflation by air, and 20 mL of intraluminal gas samples of both sites was collected through the biopsy channel. Intraluminal H2 concentrations were measured by gas chromatography. *Results*. Intragastric and intraduodenal H2 gas was detected in 566 (87.5%) and 524 (81.0%) patients, respectively. The mean values of intragastric and intraduodenal H2 gas were 8.5 ± 15.9 and 13.2 ± 58.0 ppm, respectively. The intraduodenal H2 level was increased with the progression of atrophic gastritis, whereas the intragastric H2 level was the highest in patients without atrophic gastritis. *Conclusions*. The intraduodenal hydrogen levels were increased with the progression of atrophic gastritis. It is likely that the influence of hypochlorhydria on bacterial overgrowth in the proximal small intestine is more pronounced, compared to that in the stomach.

## 1. INTRODUCTION

Breath hydrogen (H2) measurements are widely used to detect carbohydrate malabsorption [1–7]. Because bacteria represent the sole source of gut H2, fasting breath H2 gas has been used as a marker of colonic fermentation [8, 9]. If the fermentation occurs in the stomach, H2 gas should be produced and released into the gastric lumen. Gastric acid plays an important part in the prevention of bacterial colonization of the stomach and the small intestine [10, 11]. Reduction of gastric acid secretion predisposes to infection with a variety of organisms [12–14]. Intestinal bacterial overgrowth during treatment with PPI was previously reported because of an intragastric neutral pH [15–18]. Atrophic gastritis is the most common cause of reduced gastric acid secretion and *Helicobacter pylori* (*H.pylori*) seems to be the commonest cause of atrophic gastritis [19–22]. Then we attempted to collect endoscopically intraluminal gas from the stomach and the duodenum and analyze the H2 concentration in order to determine the bacterial overgrowth in the upper digestive tract.

## 2. PATIENTS AND METHODS

### 2.1. Patients

Studies were performed in 647 consecutive patients undergoing upper endoscopy, 211 men and 436 women, 19 to 85 years old (mean 60.8 ± 12.9 years). Of the patients recruited in this study, women are preponderant for one reason or another. None of the patients had a history of use of PPI, H2-receptor antagonist, antibiotics, steroids, or nonsteroidal anti-inflammatory drugs for a period of at least six months before the investigation. Twenty patients had a previous Billroth-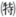 partial gastrectomy and were also excluded from analysis.

Blood samples for measurements of IgG antibody to *H.pylori* were taken prior to endoscopy. Serum samples were also examined for *H.pylori* antibody by an enzyme-linked immunosorbent assay (ELISA) using the EPI HM-CAP IgG (Enteric Products, Inc., NY) assays. All assays were performed in accordance with manufacturer's instructions. The calculated ELISA is read as positive if above 2.2.

### 2.2. Collection of intraluminal gas samples

Endoscopy was performed after a topical anesthesia gargle after a fasting period of more than 12 hours and without previous exercise. The patients were also requested to brush their teeth in the evening, but not in the morning of, the study. All patients ate meals of their own choice in the evening of the study. At the time of endoscopic examination, we intubated the stomach without inflation by air, and 20 mL of intragastric gas was collected through the biopsy channel using a 30 mL syringe. The first 5 mL was discarded for reduction of dead-space error. Once the pylorus is located, the tip of the endoscope is advanced into the descending portion of the duodenum. After that, 20 mL of intraduodenal gas was collected again by the same way. Intragastric and intraduodenal hydrogen concentrations were immediately measured by gaschromatography using Breath Analyzer TGA-2000 (TERAMECS Co., Ltd., Kyoto) and expressed in parts per million (ppm). Linear accuracy response range was 2 to 150 ppm. After collecting an intraduodenal gas sample, the endoscopist inflated the stomach by air and observed the gastric mucosa. Operators involved in the measurement of breath samples were blinded for age, sex, and endoscopic diagnosis.

### 2.3. Grading of atrophic gastritis

In this study, atrophic gastritis was classified into four stages by observing the location of the atrophic border in the stomach [23]; closed type and open type (O-1, O-2, and O-3). For closed type, the atrophic borderline is located at the lesser curvature. In the stage O-1, the atrophic borderline lies between the lesser curvature and the anterior wall of the body. In the stage O-3, the atrophic region spreads throughout the entire stomach. Stage O-2 is in-between O-1 and O-3. Stages O-1 to O-3 constitute the advanced stages of atrophic gastritis.

### 2.4. Statistical analysis

Data of intragastric and intraduodenal hydrogen were presented as mean±SD (standard deviation). Comparisons of groups were made using the unpaired *t*-test. A *P* value of <.05 was accepted as indicating statistical significance.

## 3. RESULTS

Endoscopic findings included gastric ulcer (51 patients), duodenal ulcer (24), gastric cancer (3), and gastritis (569). All of patients with gastric ulcer, duodenal ulcer, and gastric cancer had a positive result of *H.pylori* serology. Among 569 patients with gastritis, 389 were seropositive.

Over all, intragastric and intraduodenal hydrogen gases were detected in 566 (87.5%) and 524 (81.0%), respectively. The mean values of intragastric and intraduodenal hydrogen gas were 8.5 ± 15.9 (0–219) and 13.2 ± 58.0 (0–828) ppm, respectively.

Intragastric and intraduodenal H2 values and characteristics of patients in relation to endoscopic diagnosis are summarized in Table 1. The duodenal ulcer group showed a significantly younger mean age than the other groups. The intragastric H2 level was the highest in gastritis group followed by the duodenal ulcer group, and the gastric ulcer group. The intraduodenal H2 level was the highest in the gastric ulcer group among three groups.

Mean intragastric and intraduodenal H2 concentrations at different stages of atrophic gastritis are summarized in Table 2. The mean levels of intragastric H2 gas in patients with closed type, stages O-1, O-2, and O-3, were 10.5 ± 17.3 ppm, 7.4 ± 10.2 ppm, 8.0 ± 12.0 ppm, and 7.5 ± 17.0 ppm, respectively. The intragastric H2 level was the highest in patients with gastric mucosa of closed type and was significantly higher than in those with O-3 stage atrophic gastritis (*P* = .031). In contrast, the intraduodenal H2 level was the highest in patients with O-3 stage atrophic gastritis among four groups. There was a progressive increase with the progression of atrophic gastritis. The mean levels of intraduodenal H2 in patients with closed type, stages O-1, O-2, and O-3, were 7.1 ± 12.7 ppm, 4.4 ± 8.2 ppm, 8.1 ± 18.5 ppm, and 21.5 ± 86.1 ppm, respectively. The maximum of intraduodenal H2 was 828 ppm and found in 74-year-old female with O-3 stage atrophic gastritis.

## 4. DISCUSSION

Before the discovery of *H.pylori* infection in 1983 [24], many investigators reported that an increased number of bacteria had been found in the stomach in patients with achlorhydria or hypochlorhydria [25]. The type and numbers of microbial flora present in the stomach are affected by gastric pH [26–28], and a rise in intragastric pH has often been associated with an increased number of bacteria in gastric juice [29–31]. Atrophic gastritis is the most common cause of reduced gastric acid secretion. Therefore, if atrophic gastritis is closely related to the gastric and intestinal bacterial overgrowth, it is possible, we suggest, that intragastric and intraduodenal hydrogen, reflecting the fermentation by bacteria in the stomach and the duodenum, should be detected in subjects with atrophic gastritis.

The gold standard for bacterial overgrowth, against which intraluminal gas analysis must be compared, is gastric and duodenal fluid culture. Actually, the microbial flora, which is dominated by *Viridans streptococci*, *coaglase negative Staphylococci*, *Haemophilus sp*., *Neisseria spp*., *Lactobacillus spp.*, *Candida spp*., and *Aspergillus spp*. [32, 33], has been demonstrated. However, the study of gastrointestinal flora by direct methods is cumbersome, primary due to its inaccessible location. In addition, the results of identification and quantification of microbes in samples from the gastrointestinal tract are significantly influenced by difficulties in accurate tube placement, contamination during insertion, delay between sampling and inoculation of culture media, and inadequate anaerobic isolation techniques.

In the present study, of all 647 subjects, intragastric H2 was detected in 566 (87.5%) and ranged from 1 to 219 ppm, whereas intraduodenal H2 was done in 524 (81.0%), ranging from 1 to 828 ppm. This suggested that more than 80% of endoscoped patients had H2-producing bacteria in the stomach or the jejunum. Moreover, intraduodenal H2 levels were higher in patients with stage O-3 atrophic gastritis than in other groups, and there was a progressive increase with the progression of atrophic gastritis. In contrast, the intragastric H2 level was the highest in patients with gastric mucosa of closed type and was significantly higher than in those with O-3 stage atrophic gastritis. These results suggest that extensive atrophic gastritis may be more closely related to bacterial overgrowth in the jejunum, compared to that in the stomach.

Fried et al. [18] reported that most of the bacteria identified from the duodenal aspirates belonged to species colonizing the oral cavity and pharynx, suggesting a descending route of colonization. Husebye et al. [33] also reported that fasting hypochlorhydria associated with gastric colonization of microbes belonging to the oro- and nasopharyngeal flora is highly prevalent in healthy old people. At the normal acidic gastric pH, it has been thought that the stomach is sterile or contains swallowed organisms [34]. Although the pathogenesis of swallowed organisms is unknown, it is reasonable to suppose from the results of our study that these oral bacteria should continuously enter the stomach and produce H2 gas. Furthermore, it is likely that the influence of hypochlorhydria on bacterial overgrowth in the proximal small intestine is more pronounced, compared to that in the stomach.

Few studies on intragastric and intraduodenal H2 concentrations have been reported, and the clinical features and pathogenesis of intraluminal H2 gas are not clear. Bacteria represent the sole source of gut hydrogen, and H2 gas is produced at a rate of 4 L for every 12.5 g of undigested carbohydrate [35]. H2 gas is either absorbed by diffusion or consumed by bacteria to reduce carbon dioxide to methane or acetate. The intragastric H2 concentration was considered to reflect directly the intragastric fermentation and the presence of H2-producing bacteria in the stomach. Since the intragastric H2 level is not affected by absorption or metabolism of H2 unlike a breath H2 level, a trace of H2 should be detected in patients with intragastric fermentation.

In summary, unexpectedly, intragastric and intraduodenal H2 was detected in more than 80% of all subjects in this study, and the intraduodenal H2 level was increased with the progression of atrophic gastritis. Although it is unknown whether intraluminal fermentation is related to digestive diseases, a large amount of intragastric and intraduodenal H2 may cause abdominal symptoms. We have to make a further study to evaluate whether bacterial overgrowth in the stomach or the proximal small intestine is associated with some clinical symptoms or gastrointestinal diseases.

## Figures and Tables

**Table 1:** Intragastric and intraduodenal hydrogen levels in relation to endoscopic findings.

	Gastric ulcer	Duodenal ulcer	Gastritis
Number of patients	51	24	569
Age	64.5 ± 13.5	44.8 ± 10.7	61.4 ± 12.5
Male/female	34/17	18/6	156/413

Stomach (ppm)	6.1 ± 8.9	6.5 ± 12.4	8.7 ± 16.5
*P* values v.s.*	.13	.26	*
Duodenum(ppm)	22.2 ± 70.5	5.6 ± 6.8	12.8 ± 58.1
*P* values v.s.**	.12	**	.27

**Table 2:** Intragastric and intraduodenal hydrogen levels in relation to the grade of atrophic gastritis.

	Closed type	O-1	O-2	O-3
Number of patients	200	66	101	280
Age (mean ±SD)	55.3 ± 13.9	58.1 ± 13.6	59.1 ± 12.5	66.1 ± 9.8
Male/female	60/140	22/44	39/62	90/190

Stomach (ppm)	10.5 ± 17.3*	7.4 ± 10.2	8.0 ± 12.0	7.5 ± 17.0
*P* values v.s.*	*	.085	.105	.031
Duodenum (ppm)	7.1 ± 12.7	4.4 ± 8.2	8.1 ± 18.5	21.5 ± 86.1
*P* values v.s.**	.009	.055	.061	**
